# Antagonizing Effects and Mechanisms of Afzelin against UVB-Induced Cell Damage

**DOI:** 10.1371/journal.pone.0061971

**Published:** 2013-04-23

**Authors:** Seoung Woo Shin, Eunsun Jung, Seungbeom Kim, Jang-Hyun Kim, Eui-Gyun Kim, Jongsung Lee, Deokhoon Park

**Affiliations:** 1 Biospectrum Life Science Institute, Seoungnam City, Gyunggi Do, Korea; 2 Department of Dermatological Health Management, Eulji University, Seongnam, Korea; 3 Dermiskin Life Science Institute, Pyeongtaek City, Gyunggi Do, Korea; 4 ChiroChem Co., Ltd. Hannam University Science Park, Daejeon, Korea; University of Tennessee, United States of America

## Abstract

Ultraviolet (UV) radiation induces DNA damage, oxidative stress, and inflammatory processes in human keratinocytes, resulting in skin inflammation, photoaging, and photocarcinogenesis. Adequate protection of skin against the harmful effects of UV irradiation is essential. Therefore, in this study, we investigated the protective effects of afzelin, one of the flavonoids, against UV irradiation in human keratinocytes and epidermal equivalent models. Spectrophotometric measurements revealed that the afzelin extinction maxima were in the UVB and UVA range, and UV transmission below 376 nm was <10%, indicating UV-absorbing activity of afzelin. In the phototoxicity assay using the 3T3 NRU phototoxicity test (3T3-NRU-PT), afzelin presented a tendency to no phototoxic potential. In addition, in order to investigate cellular functions of afzelin itself, cells were treated with afzelin after UVB irradiation. In human keratinocyte, afzelin effectively inhibited the UVB-mediated increase in lipid peroxidation and the formation of cyclobutane pyrimidine dimers. Afzelin also inhibited UVB-induced cell death in human keratinocytes by inhibiting intrinsic apoptotic signaling. Furthermore, afzelin showed inhibitory effects on UVB-induced release of pro-inflammatory mediators such as interleukin-6, tumor necrosis factor-α, and prostaglandin-E_2_ in human keratinocytes by interfering with the p38 kinase pathway. Using an epidermal equivalent model exposed to UVB radiation, anti-apoptotic activity of afzelin was also confirmed together with a photoprotective effect at the morphological level. Taken together, our results suggest that afzelin has several cellular activities such as DNA-protective, antioxidant, and anti-inflammatory as well as UV-absorbing activity and may protect human skin from UVB-induced damage by a combination of UV-absorbing and cellular activities.

## Introduction

Ultraviolet B (UVB) exposure of the skin results in skin damage characterized by sunburn, induction of cyclobutane pyrimidine dimer (CPD) [1], immunosuppression [2], oxidative stress, and an acute inflammatory response [3], [4]. Biological systems have evolved an effective and complicated defense mechanism network to efficiently handle harmful oxidative environments [5]. Skin appears to be endowed with a variety of enzymatic antioxidants and small molecular antioxidants that inhibit oxidative damage [6]. However, the antioxidant capability of skin is often overwhelmed by overproduction of reactive oxygen species (ROS) and extensive cellular damage, which result in cell death including necrosis and apoptosis. In addition to the generation of ROS, UVB irradiation of the skin may also induce acute skin inflammation, but the use of antioxidants overcomes this imbalance. In this regard, defining novel botanical agents capable of ameliorating the adverse effects of ROS has become an important area of research, as primary prevention approaches to skin cancer have proven inadequate for lowering the incidence of skin cancer; thus, emphasizing the need to develop novel skin cancer chemopreventive agents. The use of botanicals as skin care products has recently increased to protect humans against the adverse effects of UV radiation.

Flavonoids, which are polyphenols, are exclusively produced in plants through the phenylpropanoid biosynthetic pathway to help plants combat stress such as UV irradiation and oxidative stress [7], [8]. Several lines of evidence from cell culture, animal experiments, and epidemiological studies suggest that flavonoids protect human skin from UV radiation [9]. These natural compounds show strong antioxidant effects and also show other biochemical effects in human cells, such as enzyme inhibition and anti-inflammatory and anti-carcinogenic capacities [10]. These characteristics make flavonoids potential candidates for photoprotective applications [11]–[13].

**Figure 1:**
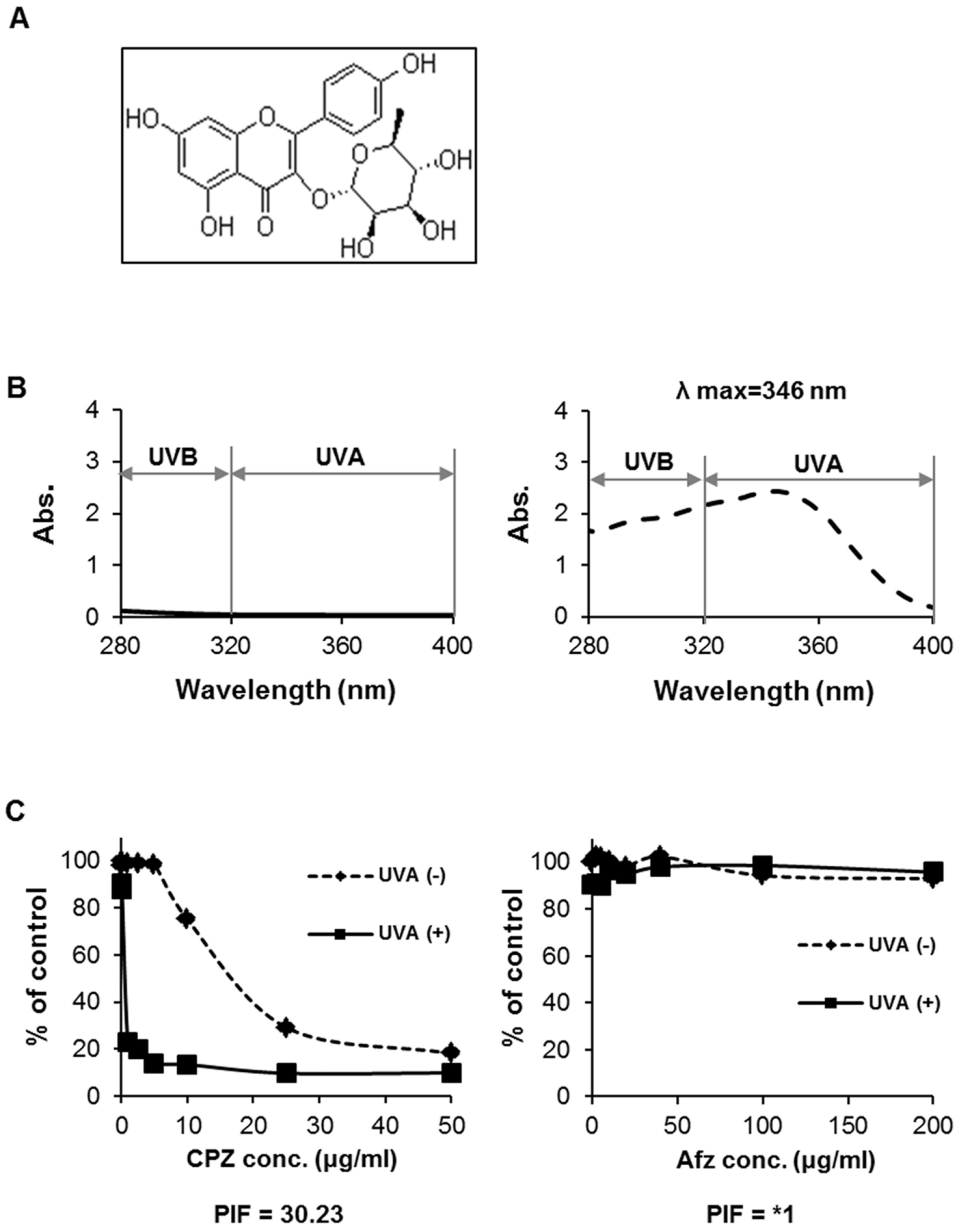
UV-absorbing properties and phototoxicity of afzelin. **A.** Chemical structure of afzelin. **B.** The UV absorbance spectra for DMSO and afzelin (dotted line). Spectra were acquired on a BioTek UV-Vis spectrometer. **C.** Phototoxicity data for afzelin and chlorpromazine (CPZ), the positive control, in the 3T3 NRU phototoxicity test. Balb/c 3T3 cells were treated with different concentrations of the tested compounds and irradiated with UVA (5 J/cm^2^). Dotted line indicates the response in the 3T3-NRU assay in the absence of UVA irradiation and the solid line indicates the response in the presence of UVA (5 J/cm^2^) irradiation. Representative data, n = 3 (B–C).

Afzelin, a flavonoid originally reported by Vareed et al., inhibits lipid peroxidation and cyclooxygenase (COX)-1 and COX-2. The structure of this compound is shown in Figure 1A. Several recent studies have indicated that afzelin inhibits the growth of breast cancer cells by stimulating apoptosis and that it is relatively non-toxic to normal cells [14]. An important implication of these findings is that this agent might play a useful role treating human skin. However, the effects of afzelin on the molecular aspects of the sunburn response in human skin cells have not been investigated.

This study was designed to assess the photoprotective effects of afzelin on UV-mediated responses in human HaCaT keratinocytes under *in vitro* conditions and an epidermal *ex vivo* equivalent model. Specifically, we determined the photoprotective effects of afzelin on UVB-mediated oxidative stress, DNA damage biomarkers, and apoptosis regulatory pathways. We found that afzelin acted by absorbing UV radiation and inhibiting the expression of proinflammatory enzymes and mediators such as COX-2 and prostaglandin E_2_ (PGE_2_).

## Materials and Methods

### Chemicals and Antibodies

The electrophoresis reagents and protein assay kit were purchased from Invitrogen (Carlsbad, CA, USA). Antibodies against cleaved poly (ADP-ribose) polymerase (PARP), cleaved caspase 3, pro-caspase 8, pro-caspase 9, p38 mitogen activated protein kinase (MAPK), phospho-p38 MAPK, extracellular regulated kinase (ERK), phospho-ERK, JNK, phospho-JNK, Bax, Bcl-xL, and Bcl-2 were obtained from Cell Signaling Technology (Beverly, MA, USA). COX-2 and β-actin were purchased from Santa Cruz Biotechnology (Santa Cruz, CA, USA). The MAPK inhibitors (SP600125 and SB203580) were acquired from Calbiochem (La Jolla, CA, USA). 5,5′,6,6′-Tetrachloro-1,1′,3,3′-tetraethylbenzimidazole carbocyanine iodide (JC-1), dihydrorhodamine 123 (DHR123), and 2′,7′-dichlorofluorescein diacetate (DCFH-DA) were purchased from Molecular Probes (Eugene, OR, USA). Propidium iodide (PI) was purchased from Sigma Chemical Co. (St. Louis, MO, USA). Afzelin (purity: 99%) was acquired from Chirochem (Daejeon, Korea). All other reagents were of analytical grade and purchased from Sigma.

### Spectophotometric Measurement

The UV/VIS spectroscopic measurements were performed on a Biotek Epoch spectrofluorophotometer (Biotek, Winooski, VT, USA) in the spectral range of 280–400 nm. Samples were measured in quartz cuvettes at a distance of 1 mm.

### Cell Culture

The HaCaT human keratinocyte cell line was purchased from CLS (Eppelheim, Germany), and cultured in Dulbecco’s modified Eagle’s medium (DMEM) supplemented with 10% fetal bovine serum, 100 units/ml penicillin and 100 µg/ml streptomycin sulfate. Balb/c 3T3 cells were obtained from the American Type Culture Collection (clone A31; Rockville, MD, USA). The culture medium used throughout these experiments was DMEM supplemented with 10% bovine calf serum and 50 units/ml penicillin and 50 µg/ml streptomycin sulfate. Cells were incubated in a humidified atmosphere of 5% CO_2_ and 95% air at 37°C. Balb/c 3T3 mouse fibroblasts were used for the phototoxicity assay.

### UV Source and Irradiation

For UV irradiation experiments, the cells were cultured on 60 mm culture dishes for 48 hr. Then, the cells were incubated with 1% serum medium for 12 h and exposed to UVB irradiation using LZC-UVB lamp (Luzchem, Ottawa, ONT, Canada), which had an emission spectrum of 280–370 nm and a peak at 312 nm, or exposed to UVA irradiation using LZC-UVA lamp (peak emission, 365 nm). The UV dose was measured with a UV light meter UV-340 (Lutron, Coopersburg, PA, USA). After irradiation, the cells were replenished with 1% serum medium including afzelin and followed for up to 12 h.

### Phototoxicity Test

The phototoxicity assay was conducted at MB Research Laboratories (Spinnerstown, PA, USA) and was based on current recommendations [15]. Briefly, Balb/c 3T3 cells were cultured in DMEM containing 10% (v/v) fetal bovine calf serum, 4 mM glutamine, and penicillin/streptomycin. Cells were seeded into 96-well microtiter plates and treated with a range of afzelin concentrations (2.5–200 µg/ml) or the chlorpromazine (0.1–50 µg/ml) positive control. The cultures were treated for 1 h at 37°C prior to irradiation. One set of plates was exposed to 5 J/cm^2^ UVA, and a second set of plates was kept in the dark for the same period. After irradiation, the medium was aspirated from each well, and the cells were washed with phosphate-buffered saline. Finally, 0.2 ml medium was added to each well, and the plates were further incubated for 24 h at 37°C in a humidified atmosphere of 5% CO_2_ in air. At the end of the incubation, cell viability was assessed by measuring Neutral Red uptake for 3 h [16]. Cell viability is expressed as a percentage of untreated solvent controls and was calculated for each test concentration. The concentration responses obtained in the presence and absence of irradiation were compared at the half-maximal response concentration (EC_50_) level to predict phototoxic potential. The photoirritancy factor (PIF) was determined:
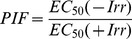



If both EC_50_ (−Irr) and EC_50_ (+Irr) could not be calculated due the lack of cytotoxicity at the highest tested concentration, no phototoxic potential was indicated. In this case, “PIF = *1” was used as the result:
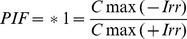



### Analysis of DNA Damage by the Comet Assay

UVB-induced DNA damage on a per cell basis was determined using the comet assay, as described previously [17]. HaCaT cells pre-treated with afzelin (0–200 µg/ml) for 1 h or vehicle-treated HaCaT cells were exposed to UVB (20 mJ/cm^2^) and harvested 1 h later for the comet assay. Briefly, the cells were harvested and re-suspended in ice cold PBS after UVB treatment. Approximately 1×10^4^ cells in 80 µL of 0.5% (w/v) low melting point agarose were pipetted onto a frosted glass slide coated with a thin layer of 1.0% (w/v) agarose, covered with a coverslip, and placed on ice for 10 min. The cover slip was removed and the slides were immersed in ice-cold lysis solution containing 2.5 M NaCl, 10 mM Tris, 100 mM Na_2_-EDTA, and 1% (w/v) N-lauroyl-sarcosine, pH 10.0, and 1.0% Triton X-100 was added immediately before use. After 2 h at 4°C, the slides were placed into a horizontal electrophoresis tank filled with buffer (0.3 M NaOH, 1 mM EDTA, pH 13) and subjected to electrophoresis for 30 min at 300 mA. The slides were transferred to neutralization solution (0.4 M Tris-HCl) for 3×5 min washes and stained with ethidium bromide for 5 min. The comets were examined and photographed using a fluorescence microscope. Slides were viewed using the 20× objective of an Evos fluorescent microscope equipped with epifluorescence optics (Advanced Microscopy Group, Bothell, WA, USA). The tail lengths (µm) of a minimum of 50 comets in each sample were analyzed using software imaging analysis (Comet assay IV; Perceptive Instruments, Bury St. Edmunds, UK). The length of the comet was quantified as the distance from the centrum of the cell nucleus to the tip of the tail in pixel units, and tail length was expressed as mean ± standard deviation (SD) from 50 comets.

### Cyclobutane Pyrimidine Dimer (CPD) Quantification

DNA was isolated using DNAzol reagent (Invitrogen). DNA concentration was measured on a BioTek Epoch spectrophotometer (Biotek,) and the CPDs were quantified with an Oxiselect Cellular UV-induced DNA damage ELISA kit from Cell Biolabs (San Diego, CA, USA), according to the manufacturer’s instructions.

### Immunofluorescence

HaCaT cells were seeded on 96-well black plate and treated for 1 h with afzelin as described above. The cells were fixed in formaldehyde 1 h after irradiation (20 mJ/cm^2^ UVB) and rinsed with a combined blocking/permeabilization HCA immunofluorescence buffer. A primary antibody solution (1∶500 dilution p-p53 S15; 1∶800 dilution γ-H2AX) was added to each well, followed by a 1 h incubation at room temperature. The wells were rinsed, and the secondary antibody conjugated with Alexa Fluor 488/PI nuclear counterstain solution was added to each well and incubated in the dark for 1 h at room temperature. The wells were rinsed and sealed prior to HCA imaging. Plates were imaged on a GE IN Cell Analyzer 1000, and the images were analyzed with GE IN Cell Analyzer1000 Workstation software. The number of Alexa Fluor 488-positive cells/100 PI-positive cells was determined in two individual high-power fields per experiment by two independent assessors.

### Intracellular ROS Measurement

Two assays were used to measure ROS. Cells pre-loaded with 10 µM H_2_DCFDA for 30 min were exposed to UVB, and were then lysed in 0.1% Triton X-100. The fluorescence intensity of each lysate was measured at an excitation wavelength of 485 nm and an emission wavelength of 530 nm using a fluorometer (INFINITE M200, Tecan, Männedorf, Switzerland). The second method used cells that were pre-loaded with 10 µM H_2_DCFDA for 30 min and exposed to UVB. The cells were observed immediately under an Evos fluorescent microscope [18].

### Lipid Peroxidation Assay

The cells were harvested and washed PBS, and microsomal fraction was prepared as described earlier [19]. Briefly, 0.2 ml of the microsomal fraction was treated with 0.2 ml of 8.1% SDS and 3 ml thiobarbituric acid. Total volume was made up to 4 ml with distilled water and kept at 95°C in water bath for 1 h. Color was extracted with n-butanol and pyridine (15∶1 v/v). The absorbance was measured at 530 nm, and the resultant lipid peroxidation was expressed in terms of percentage of control.

### Cell Viability Assay

The cytotoxicity of afzelin after UVB irradiation was determined using 3-(4,5-dimethylthiazol-2-yl)-2,5 diphenyltetrazolium bromide (MTT) reduction to the corresponding blue formazan by viable cells. Cells were grown to ∼80% confluence and maintained in 1% serum medium for 12 h prior to UV exposure. The level of blue formazan was measured spectophotometrically and used as an indirect index of cell density. Briefly, cells were exposed to MTT (1 mg/ml) for 3 h at 37°C. The medium was removed, and the cells were solubilized with dimethyl sulfoxide. After complete solubilization, the presence of blue formazan was evaluated spectrophotometrically by measuring absorbance at 540 nm (reference, 620 nm) with an enzyme-linked immunosorbent assay (ELISA) plate reader. Viability was expressed as a percentage of the control.

### Flow Cytometry

Both adherent and floating cells were collected, washed with ice-cold PBS, and fixed with 70% ice-cold ethanol overnight at 4°C 12 h following UVB irradiation and/or afzelin treatment. Fixed cells were washed twice with PBS and treated with 100 µg/mL RNase for 30 min at 37°C and then stained with 1 mg/ml PI in PBS containing 0.05% Nonidet-P40. The cells were then analyzed with a FACScan flow cytometer (Becton Dickinson, Franklin Lakes, NJ, USA). The percentages of cells in different cell cycle phases were evaluated from an analysis of DNA histograms. Cells with a sub-G_0_/G_1_ DNA (sub-G_1_) were considered apoptotic cells.

### DNA Fragmentation Assay

Oligonucleosomal DNA fragmentation was identified by agarose gel electrophoresis. To determine the degradation of chromosomal DNA into nucleosome-sized fragments, a 500-µl aliquot of the lysis buffer (100 mM Tris-HCl, pH 8.5, 5 mM EDTA, 0.2 M NaCl, 0.2% SDS, and 0.2 mg/ml proteinase K) was added to the cell pellet (2×10^5^ cells) and incubated at 37°C overnight. DNA was obtained by ethanol precipitation, separated in a 0.8% agarose gel, and visualized under UV light.

### Western Blot Analysis

Cells were washed twice with cold PBS and lysed in 150 µl of sample buffer (100 mM Tris–HCl, pH 6.8, 10% glycerol, 4% SDS, 1% bromophenol blue, and 10% β-mercaptoethanol). The proteins were resolved on a NuPAGE Novex 10% Bis-Tris Gel (Invitrogen). Following electrophoretic transfer of the proteins onto nitrocellulose membranes, they were subsequently hybridized with primary antibody (1∶1000) followed by a horseradish peroxidase-conjugated secondary antibody (1∶2000). Finally, the protein bands were visualized using the PowerOpti-ECL Western Blotting Detection reagent (Anigen, Hwaseong, Korea). Protein bands were quantified with Image J software.

### Measurement of Apoptotic Sunburn Cells in Reconstructed Skin

The epidermal equivalent MelanoDerm which is made using normal human keratinocytes and melanocytes obtained from Asian neonatal foreskin tissue, was purchased from MatTek Corp. (Ashland, MA, USA). MelanoDerm was grown at the air/liquid interface of MEL-NHM-113 maintenance medium (MatTek). Some epidermal samples were topically treated with 100 µg/ml afzelin 12 h before UVB exposure. Epidermal equivalents were fixed in paraformaldehyde, embedded in paraffin, and sections were cut using standard techniques. The sections were deparaffinized in xylene and hydrated through a graded ethanol series. Skin sections were stained conventionally with hematoxylin and eosin (H&E) to identify sunburned cells as described previously [20]. Apoptotic cells are morphologically distinct due to cell shrinkage and nuclear condensation that stains darker with H&E. Dark stained cells were scored in five random fields/sample and the percentage/field was calculated.

### Terminal Deoxynucleotidyl Transferase dUTP Nick End Labeling (TUNEL) Staining of Apoptotic Cells

Apoptotic cells were detected using the DeadEnd Colorimetric TUNEL System (Promega, Madison, WI, USA), following the manufacturer’s protocol with some modifications. Briefly, deparaffinized and rehydrated tissue sections were permeabilized with 30 mg/ml Proteinase K for 1 h at 37°C. Then, the sections were quenched of endogenous peroxidase activity in 3% hydrogen peroxide for 10 min. After a thorough washing with 1× PBS, the sections were incubated with equilibration buffer for 10 min, and then the TdT reaction mixture was added to the sections, except for the negative control, and incubated at 37°C for 1 h. The reaction was stopped by immersing the sections in 2× saline-sodium citrate buffer for 15 min. Streptavidin-HRP (1∶500) was added to the sections for 30 min at room temperature, and after repeated washings, sections were incubated with substrate DAB until the color developed (5–10 min). The sections were mounted after rehydration and observed for TUNEL-positive cells.

### Mitochondrial Redox Status and Damage

The JC-1 fluorescent probe was used to estimate mitochondrial membrane potential. JC-1 (10 µM) was added to the cells and incubated at 37°C for 30 min. The ratio of the intensity of green fluorescent monomers to the intensity of JC-1 aggregates is directly related to mitochondrial membrane potential [21]. The levels of mitochondrial hydrogen peroxide were determined with the DHR 123 oxidant-sensitive fluorescent probe using a fluorescent fluorometer [18]. Cells pre-loaded with 10 µM DHR123 for 30 min were exposed to UVB and were then lysed with 0.1% Triton X-100. The fluorescence intensity of each lysate was measured at an excitation wavelength of 485 nm and an emission wavelength of 530 nm using a fluorometer (INFINITE M200).

### Cytochrome *c* ELISA

An ELISA kit was used to quantify cytochrome *c* in subcellular fractions (R&D Systems, Minneapolis, MN, USA). After pretreatment with UVB (20 mJ/cm^2^), HaCaT cells (6×10^6^) were incubated for 12 h with afzelin (40–200 µg/ml), harvested with trypsin, centrifuged briefly at 800×g, and the supernatant was discarded. The cell pellet was resuspended and washed with PBS. Cells were pelleted at 1,000×g for 5 minutes, and the supernatant was discarded. The cell pellet was resuspended with Digitonin Cell Permeabilization Buffer (250 mM sucrose, 137 mM NaCl, 70 mM KCl, 4.3 mM Na_2_HPO_4_, 1.4 mM K_2_HPO_4_, 0.2 mg/mL digitonin and 0.1% Hydorol M), vortexed, and incubated on ice for 5 minutes. The cells were then centrifuged at 1000×g for 5 minutes at 4°C. The supernatants were saved, as they contained the cytosolic fraction with cytochrome *c*. The remaining pellet was resuspended in RIPA Cell Lysis Buffer 2 (50 mM Tris HCl, pH 7.4, 150 mM NaCl, 1 mM EDTA, 1 mM EGTA, 1% Triton X-100, 1% sodium deoxycholate, and 0.1% sodium dodecyl sulfate (SDS)), vortexed, and incubated on ice for 5 minutes. The lysate was centrifuged at 10,000×g for 10 minutes at 4°C, and the supernatant (mitochondrial fraction) was collected and protein was quantified. The samples were then treated with a conjugate reagent, transferred to microwell strips coated with anti-cytochrome *c* antibody, and incubated for 60 minutes at room temperature. The samples were then treated with a peroxidase substrate reagent and incubated for 15 minutes at room temperature. After adding a stop solution (0.5 M H_2_SO_4_), the optical density of each well was measured at 450 nm. The cytochrome *c* concentration was determined from a standard curve. Fractions were run in the assay and the resulting picogram determinations were divided by the protein concentration. The resulting values are expressed as pg/mg of total protein from each fraction.

### Inflammatory Cytokine Assay

The cells were irradiated with the indicated UVB doses and then incubated with the indicated afzelin concentration for 12 h. After 12 h, TNF-α and IL-6 concentrations in the culture supernatant were measured using ELISA kits (R&D Systems), according to the manufacturer’s instructions. Briefly, culture supernatants were added to 96-well plates, and diluted biotinylated TNF-α or IL-6 was added to the sample wells. The samples were incubated at room temperature for 3 h, after which the wells were washed. Streptavidin-HRP was distributed to the sample wells and the plate was incubated for 30 min at room temperature. The wells were washed, and 3, 3′, 5, 5′-tetramethylbenzidine substrate solution was added. Finally, the samples were incubated in the dark, and absorbance was read at 450 nm.

PGE_2_ concentrations in the supernatants were analyzed with a high sensitivity PGE_2_ ELISA (R&D Systems). Culture supernatants were added to 96-well plates and alkaline phosphatase-conjugated PGE_2_ antibodies were added to the sample wells. The samples were incubated at room temperature for 2 h. Sample wells were washed, and p-nitrophenyl phosphate (pNpp) substrate solution was added. Finally, the samples were incubated at room temperature for 1 h, and absorbance was read according to the manufacturer’s instructions.

### Statistical Analysis

All data are expressed as mean ± SD. Differences between the control and treatment group were evaluated by Student’s *t-*test using Statview software (Abacus Concepts, Piscataway, NJ, USA). A *P*<0.01 was considered statistically significant.

## Results

### UV-absorbing Properties and Photosafety of Afzelin

UV irradiation at the surface of the earth has wavelengths between 290 nm and 400 nm. Spectrophotometric measurements with 1% (v/v) solubilized afzelin distributed on a 1-mm glass plate revealed the afzelin extinction profile, with absorption maxima in the UVB (290–320 nm) and UVA (320–400 nm) ranges (Figure 1B). UV transmission at <376 nm was <10% (data not shown). These data indicate that afzelin may prevent photons from entering the skin when it is applied topically.

The 3T3 NRU phototoxicity test has been established as an alternative *in vitro* methodology to various *in vivo* phototoxic evaluations [22]. This assay is the only photosafety test that has been recommended by OECD guidelines and has been validated by international standards (OECD guideline, 2004). The test assesses the cytotoxic effects of UVA-irradiated compounds on a Balb/c 3T3 mouse fibroblast cell line using a concentration-dependent reduction in Neutral Red uptake. In this investigation, the cell viability curves of test compounds using cells that were and were not exposed to irradiation were determined up to the indicated concentrations. Figure 1C shows representative cell viability curves for 3T3 cells after exposure to afzelin and chlorpromazine (positive control). Cytotoxicity was not observed with/without UVA irradiation at the tested afzelin concentration. Therefore, the PIF values of the afzelin-treated group with/without UVA irradiation were estimated to be *1. In contrast, cytotoxicity occurred at a lower chlorpromazine concentration after exposure to UVA light, resulting in enhanced chlorpromazine-induced cytotoxicity. The PIF value of chlorpromazine was 30.23. OECD guideline classification criteria based on PIF values are defined in three groups, including phototoxic molecules (PIF>5), mildly or probably phototoxic molecules (2<PIF<5), and non-phototoxic molecules (PIF<2) (OECD guideline, 2004). Thus, afzelin was considered a non-phototoxic compound.

### UVB-induced DNA Damage is Attenuated by the UV-absorbing Activity of Afzelin Itself

**Figure 2:**
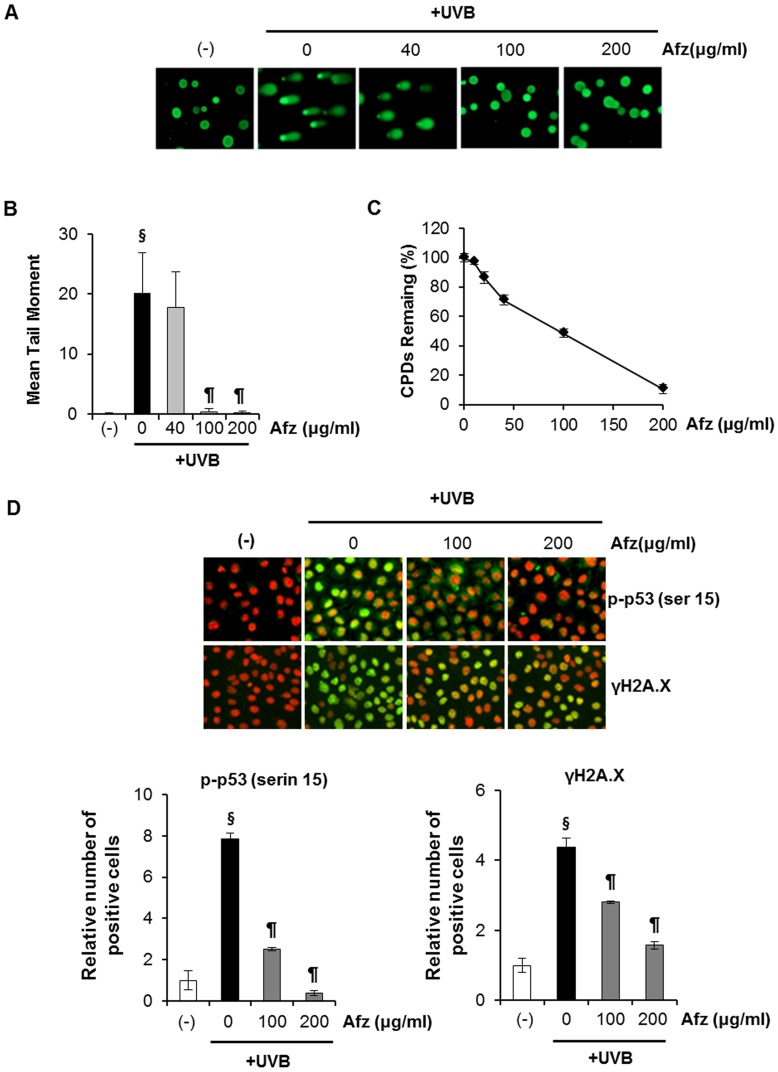
UVB-induced DNA damage is attenuated by the UV-absorbing activity of afzelin itself. Cells were treated with different concentrations of afzelin (40–200 µg/ml) for 1 h before being exposed to UVB (20 mJ/cm^2^). **A.** At 30 min after UVB irradiation, the percentage of cellular DNA damage was detected by the comet assay after rewinding the DNA with alkaline buffer. Slides were stained with SYBR and viewed under a fluorescence microscope. Representative data, n = 3 **B.** Tail lengths (µm) of a minimum of 50 comets in each sample were analyzed using software image analysis. **C.** Enzyme-linked immunosorbent assay (ELISA) analysis of the percentage (%) of cyclobutane pyrimidine dimers (CPD) remaining in cells pretreated with vehicle or different concentrations of afzelin (40–200 µg/ml) at 30 min before UVB irradiation (20 mJ/cm^2^). **D.** Immunofluorescence analysis of phosphoactive histone H2A.X (γH2A.X; green) and phospho-p53 (serin 15) (green). Staining with propidium iodide (PI; red) was performed to observe cell nuclei. The number of Alexa Fluor 488-positive cells per 100 PI-positive cells was determined in two individual high-power fields per experiment by two independent assessors. Images were analyzed with GE IN Cell Analyzer1000 Workstation software. Data are presented as means ± SD, n = 3 (B-D). ^§^ *P*<0.01 compared with the vehicle-treated group, ^¶^ *P*<0.01 compared with the UVB treated group (B–D).

A neutral pH comet assay has been used to assess the presence of double-strand breaks in UV-treated cells [23]. This assay involves lysis of cells in agarose at neutral pH and separation of the DNA fragments from the cells by electrophoresis to form single cell “comets” that are observed microscopically. The size of the comet “tails” is proportional to the extent of DNA breakage. We determined and verified the photoprotective effect of afzelin on UVB-induced cellular DNA damage using the comet assay, which was also used as an apoptosis biomarker. In these experiments, afzelin was treated before UV irradiation to confirm UV-absorbing activity of afzelin. As shown in Figure 2A and 2B, exposure of HaCaT cells to UVB radiation (20 mJ/cm^2^) resulted in extensive DNA damage, as reflected by the comet tail length, compared to cells that were not exposed to UVB radiation. However, treating cells with afzelin (40–200 µg/mL) before UV irradiation resulted in a reduced amount of DNA damage compared to cells not treated with afzelin but exposed to UVB, as evidenced by the comet structure (Figure 2B). The DNA damaging effect and its prevention by afzelin were determined by measuring comet tail length under a microscope. The tail length data (µm) are shown as mean ± SD from at least 50 cells or comets in each treatment group (Figure 2B). The results suggest that afzelin reduced UVB-induced DNA damage compared to that in UVB alone-exposed control cells by absorbing UVB.

CPD represent major UVB-induced DNA damage. Therefore, CPD formation was determined to evaluate the sunscreening effect of afzelin. As shown in Figure 2C, UVB irradiation strongly induced CPD formation, and this effect was markedly suppressed by afzelin treatment before UVB irradiation. UVB-induced generation of CPD occurs as an immediate event, and CPDs are detectable shortly after UVB exposure. Afzelin clearly prevented CPD formation by absorbing the UV radiation, because reduced CPD formation was apparent at 1 h after UVB irradiation before DNA repair mechanisms could occur.

The kinetics of UVB-induced DNA damage were investigated by analyzing phospho-p53 (Ser-15) and γ-H2AX expression levels. UVB irradiation induced p53 (Ser-15) and γ-H2AX phosphorylation in a dose-dependent manner in HaCaT keratinocytes (data not shown). In addition, we determined the intracellular location of phospho-p53 (Ser-15) and γ-H2AX proteins in HaCaT keratinocytes using immunofluorescence staining and confocal microscopy. The intensity of green fluorescence (FITC) shows the phospho-p53 (Ser-15) (Figure 2D, upper image) and γ-H2AX proteins (Figure 2D, below image); this intensity increased in nuclei after UVB irradiation. However, green fluorescence intensity was significantly lower in cells pretreated with afzelin when compared to that in nuclei of untreated cells when HaCaT cells were exposed to UVB (Figure 2D).

### Afzelin Suppresses the Increase in Intracellular ROS Induced by UVB Radiation

**Figure 3:**
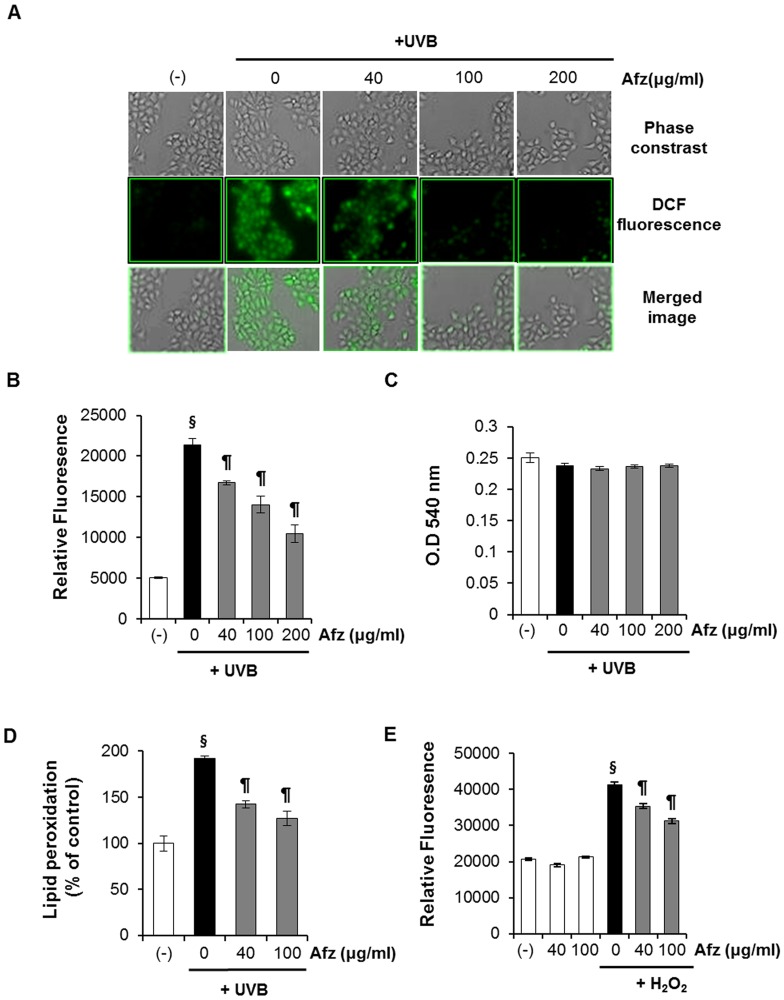
Radical scavenging effect of afzelin which was treated after UV irradiation. **A–D.** Effects of afzelin on UVB-induced reactive oxygen species (ROS) production in HaCaT cells. CM-H_2_DCFDA (5 µM) was added, and the cells were incubated for 20 min in the dark. The cells were then washed and irradiated with 20 mJ/cm^2^ UVB. The cells were treated with afzelin for 1 h. Subsequently, **A.** DCF fluorescence was visualized by fluorescence microscopy. The average fluorescence intensity values were calculated using Image J software. Representative data, n = 3 **B.** ROS-induced DCF formation was measured using a spectrophotometer. **C.** Cell viability was measured 1 h after UVB irradiation. **D.** Effects of afzelin on UVB-mediated lipid peroxidation in HaCaT cells. HaCaT cells were treated with afzelin (40–100 µg/ml) for 12 h after being exposed to UVB (20 mJ/cm^2^) radiation. At 12 h after UVB irradiation, the cells were processed for lipid peroxidation. **E.** Effect of afzelin on ROS production in H_2_O_2_-treated HaCaT cells. The cells were treated as described in **A** but incubated for 30 min with 1 mM H_2_O_2_ instead of receiving UVB irradiation. Data are means ± SD, n = 3 (B-E). ^§^ *P*<0.01 compared with the vehicle-treated group, ^¶^ *P*<0.01 compared with the UVB or H_2_O_2-_treated group (B–E).

Until now, we found that UV-induced DNA damage was reduced by UV-absrobing activity of afzelin. As a next step, we investigated cellular activities of afzelin except UV-absorbing activity. To this end, afzelin was treated after UV irradiation to exclude the UV-absorbing properties of afzelin. Intracellular ROS generation was monitored to investigate the effects of afzelin on UVB-induced oxidative stress in HaCaT cells. Afzelin was added to the cells after UVB irradiation. The antioxidative activity of afzelin was determined with the oxidant-sensitive fluorescent dye DCFH-DA in UVB-irradiated HaCaT cells. The UVB-induced increase in ROS generation decreased significantly in a concentration-dependent manner following afzelin treatment (Figure 3A and 3B). Fluorescence microscopy showed that 100 µg/ml afzelin completely inhibited formation of the DCF green fluorescent dye (Figure 3A). A 20 mJ/cm^2^ UVB dose was used in the ROS experiments. No effect on cell viability of afzelin was observed either without or in combination with UVB (Figure 3C). Next, we evaluated the effects of afzelin on UVB-mediated lipid peroxidation, which is a well-accepted oxidative stress marker. As shown in Figure 3D, UVB irradiation of cells resulted in a significant 192% increase in lipid peroxidation at a UVB dose of 20 mJ/cm^2^ when compared to that in untreated cells. Treating the cells with afzelin significantly inhibited UVB-mediated increased lipid peroxidation by 50 and 65% at afzelin concentrations of 40 and 100 µg/ml, respectively.

We also investigated the antioxidant properties of afzelin in H_2_O_2_-treated cells. H_2_O_2_ is generated in human skin by UVB irradiation. H_2_O_2_ is transformed within the skin to other ROS [24]. Afzelin showed concentration-dependent antioxidant effects (Figure 3E). These results suggest that afzelin significantly inhibited UVB-induced oxidative stress in HaCaT cells, as directly evidenced by the reduction in intracellular ROS.

### Afzelin Inhibits UV-induced Cytotoxicity in HaCaT Cells

**Figure 4:**
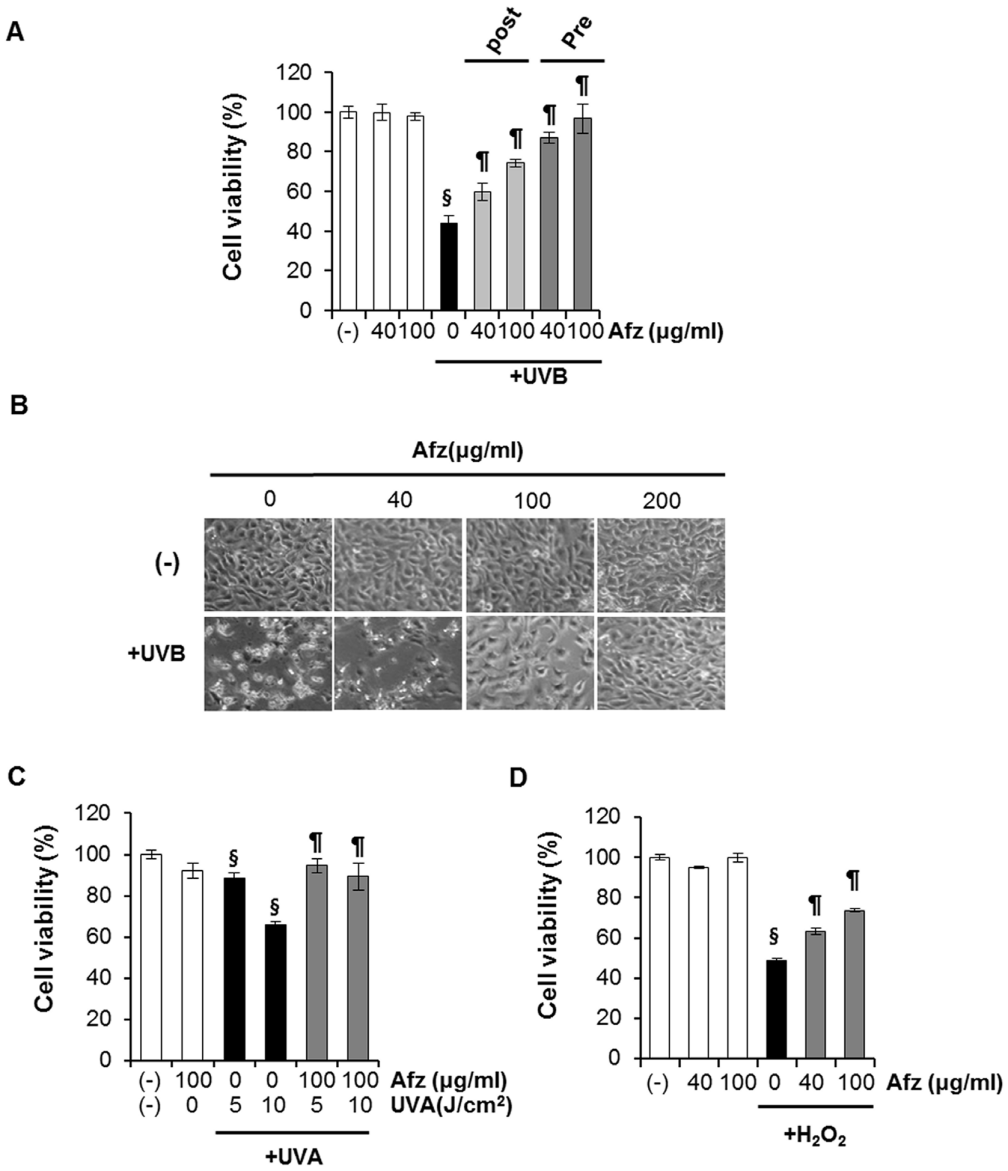
Afzelin treatment protects HaCaT cells against UVA, UVB, and H_2_O_2_-mediated decreased cell viability. HaCaT cells were treated with different concentrations of afzelin (40–200 µg/ml) for 12 h after being exposed to oxidative stress (UVA, UVB, and H_2_O_2_). **A.** The percentage of viable cells was assessed using the MTT assay at 12 h after UVB irradiation. **B.** Phase-contrast microscopy. HaCaT cells were irradiated with UVB (20 mJ/cm^2^) and then incubated without or with afzelin (40–200 µg/ml) for 12 h. A representative picture from three independent experiments with similar results is shown. **C.** The cells were treated as described above but irradiated with UVA (5–10 J/cm^2^) instead of UVB irradiation. **D.** The cells were treated as described above but incubated for 12 h with 1 mM H_2_O_2_ instead of UVB irradiation. Data are presented as means ± SD, n = 5 (A,C and D). ^§^ *P*<0.01 compared with the vehicle-treated group, ^¶^ *P*<0.01 compared with the UVB or H_2_O_2-_treated group (A,C and D).

We initially investigated the effect of afzelin (40–200 µg/ml) on the UVB-mediated decrease in cell viability. As expected, UVB (20 mJ/cm^2^) irradiation of HaCaT cells resulted in decreased cell viability. This UVB-induced cell growth inhibition was significantly attenuated by treatment (pre- or post-treatment) of cells with afzelin (40–200 µg/ml) in a concentration-dependent manner as determined by the MTT assay (Figure 4A). UVB (20 mJ/cm^2^) irradiation caused the cells to become round and detached from the surface of the plate, whereas afzelin treatment prevented these morphological changes, as assessed by phase contrast microscopy (Figure 4B). Afzelin treatment of control cells resulted in no cytotoxic effects. Next, we evaluated the effect of afzelin against the UVA-mediated decrease in cell viability. We selected an afzelin concentration of 100 µg/ml, as this concentration provided substantial protection against the UVA-mediated decrease in cell viability (Figure 4C). We also tested the protective properties of afzelin in H_2_O_2_-treated cells. Afzelin showed concentration-dependent protective effects (Figure 4D).

### Afzelin Protects HaCaT Cells from UVB-mediated Apoptosis

**Figure 5:**
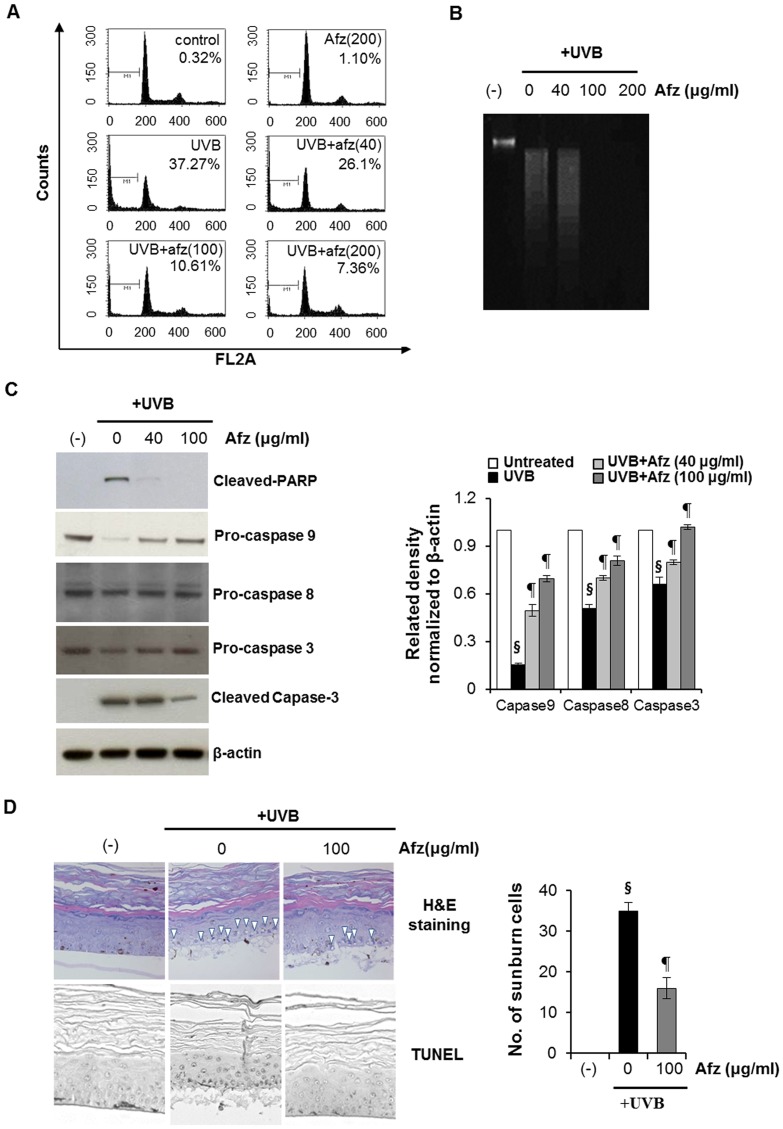
Inhibitory effect of afzelin on UVB-induced apoptosis. HaCaT cells were treated with afzelin (40–200 µg/ml) for 12 h after irradiation with 20 mJ/cm^2^ UVB. **A.** Cell cycle analysis with cellular DNA content was examined by flow cytometry. The sub-G_1_ region (presented as “M1”) includes cells undergoing apoptosis. The number in each panel refers to the percentage of apoptotic cells. **B.** Agarose gel electrophoresis of HaCaT cell nuclear DNA fragments exposed to UVB (20 mJ/cm^2^) **C.** Immunoblot analysis of various apoptosis-related proteins in HaCaT cells unexposed or exposed to UVB (20 mJ/cm^2^). Cell extracts were subjected to 10–12.5% sodium dodecyl sulfate-polyacrylamide gel electrophoresis (SDS-PAGE) and immunoblotted with antibodies against cleaved caspase-3, cleaved poly(ADP-ribose) polymerase (PARP), procaspase-8, and procaspase-9. β-Actin was included as an internal control. Immunoblot bands were quantified using Image J software**.** Representative data, n = 5 (A–C). **D.** Reconstructed skin was exposed to UVB (20 mJ/cm2) and then treated for 12 h with or without 100 µg/ml afzelin. Skin specimens were removed, fixed, paraffin-embedded, and processed for analysis by hematoxylin and eosin (H&E) staining and for an analysis of apoptotic cells by terminal deoxynucleotidyl transferase dUTP nick end labeling (TUNEL). Sunburn cells (arrowheads point to some examples) were calculated as the mean of five randomly selected fields (400×) from each skin sample. Data are means ± SD, n = 5 (C-D). ^§^ *P*<0.01 compared with the vehicle-treated group, ^¶^ *P*<0.01 compared with the UVB treated group (C–D).

We examined whether the protective effect of afzelin on the UVB-mediated decrease in cell viability was due to inhibiting apoptosis. The extent of apoptosis was quantified by flow cytometry analysis of afzelin-treated and -untreated cells exposed to a 20 mJ/cm^2^ dose of UVB. As shown in Figure 5A, UVB irradiation of cells resulted in 37.27% apoptotic cells at a 20 mJ/cm^2^ UVB dose. However, pre-treatment of afzelin resulted in only 10.61 and 7.63% apoptotic cells at afzelin concentrations of 100 and 200 µg/ml, respectively. Afzelin itself did not induce apoptosis at these concentrations. We found that treating HaCaT cells with afzelin after UVB irradiation prevented UVB-mediated apoptosis. Next, UV-triggered apoptosis in HaCaT cells was determined by measuring DNA using agarose gel electrophoresis (Figure 5B). DNA fragmentation decreased significantly in afzelin-treated cells after exposure to UVB compared to that in untreated cells. We also evaluated changes in apoptotic marker proteins as a result of UVB irradiation and the influence of afzelin on these proteins. Previous studies have identified caspases as important apoptosis mediators induced by a range of stimuli [25]. Activation of capase-3, -8, and -9 in HaCaT cells was assessed by immunoblot analysis of lysates from cells that had been exposed to UVB, with and without afzelin treatment. As expected, we found that UVB irradiation resulted in decreased procaspase-3,-8, and -9 protein expression, indicating that the proform of caspase was cleaved into the active form. However, the cleavage was significantly reduced by afzelin treatment after UVB irradiation (Figure 5C). These results indicate that treating cells with afzelin after UVB irradiation inhibited the UVB-mediated decrease in procaspase-3, -8, and -9 protein expression, as demonstrated by relative density and Western blot analysis. UVB irradiation also induced the formation of PARP proteolytic cleavage fragments, indicating imminent apoptosis. The cleaved PARP product markedly increased in untreated cells compared to that in afzelin-treated cells after exposure to UVB (Figure 5C). Taken together, UVB induced procaspase-3 cleavage into the active caspase-3 form, and caspase-3 induced PARP degradation. The results also indicate that afzelin exhibited a protective effect on UVB-induced apoptosis. Using TUNEL staining, we found that a topical application of 100 µg/mL afzelin after UVB irradiation prevented UVB-induced apoptosis in epidermal equivalent models. Characteristic dyskeratotic sunburned cells with pyknotic nuclei were detected by histomorphological analysis using H&E staining. In parallel with the TUNEL assay, H&E staining of apoptotic sunburned cells increased markedly in UVB-exposed epidermal equivalent models. But, the afzelin-treated groups after UVB irradiation showed a significant reduction in sunburned cells, accounting for a 55% inhibition (Figure 5D). These results indicate that in addition to the UV-absorbing properties, afzelin has cellular activities to prevent UV-induced apoptosis in epidermal keratinocytes both *in vitro* and *ex vivo*.

### Afzelin Modulates the Mitochondrial Pathway in UVB-induced Apoptosis of HaCaT Cells

**Figure 6:**
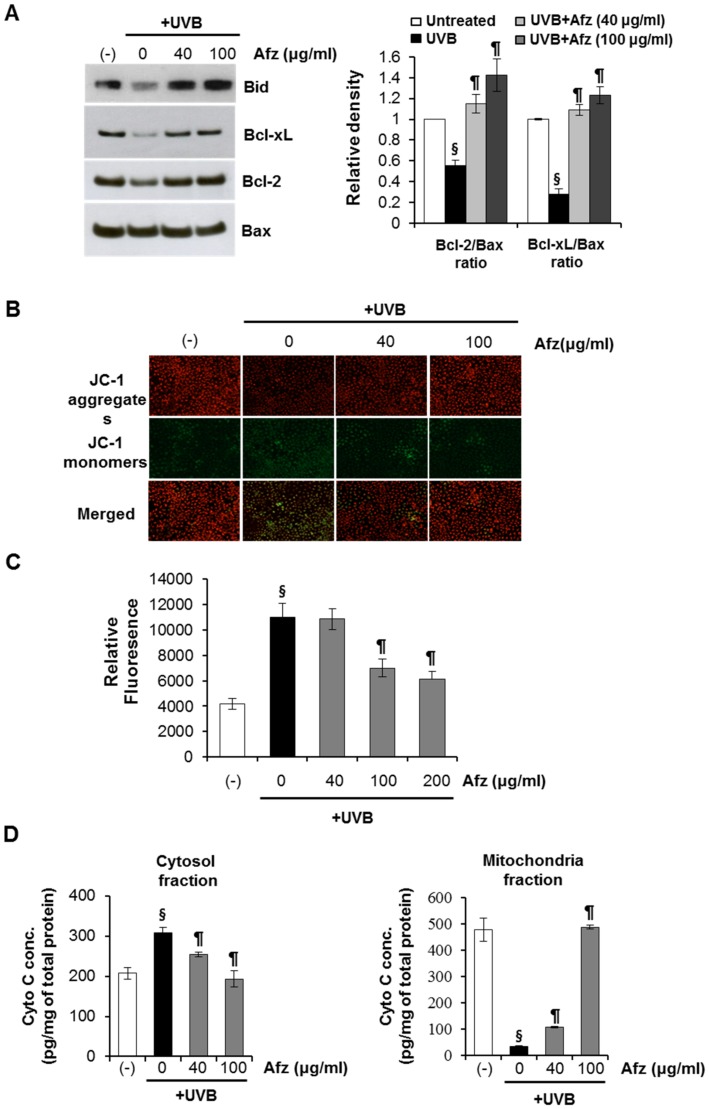
Afzelin inhibits UVB-induced mitochondrial effects. **A.** Effect on UVB mediated changes on bid, Bcl-xL, Bcl-2, and Bax protein expression. HaCaT cells were irradiated with UVB (20 mJ/cm^2^) and incubated for 12 h. Cell lysates were subjected to immunoblot analysis with antibodies against bid, Bcl-xL, and Bax. The Bcl-xL/Bax and Bcl-2/Bax ratios are presented in graphs. **B.** Mitochondrial membrane potential (Δψ_m_) was measured by staining HaCaT cells with JC-1 followed by fluorescence microscopy analysis. HaCaT cells were incubated with the JC-1 probe for 30 min. Mean (red/green) fluorescence, expressed as a percentage of the control, indicates the ratio of high/low mitochondrial membrane potential. Representative data, n = 3 (A–B). **C.** DHR 123 was employed to detect mitochondrial hydrogen peroxide. Reactive oxygen species (ROS)-induced DHR 123 fluorescence was measured using a spectrophotometer. **D.** Enzyme-linked immunosorbent assay (ELISA) analysis of cytochrome *c* in cytosolic and mitochondrial fractions of HaCaT cells exposed to UVB (20 mJ/cm^2^). HaCaT cells were treated with afzelin (40–100 µg/ml) for 12 h after exposure to UVB (20 mJ/cm^2^) radiation. Then, the cells were processed for cytochrome *c* analysis. Data are means ± SD, n = 3 (A–D). ^§^ *P*<0.01 compared with the vehicle-treated group, ^¶^ *P*<0.01 compared with the UVB _-_treated group (A–D).

We examined expression of apoptosis-related Bcl-2 family proteins to further understand the anti-apoptotic mechanisms of afzelin against UVB-induced apoptosis in HaCaT cells. Bid, a death agonist member of the Bcl-2/Bcl-xL family, is a specific proximal caspase-8 substrate in the Fas signaling pathway [26]. UVB irradiation of HaCaT cells resulted in cleavage of the bid protein 12 h after UVB exposure, but treatment with afzelin after UVB irradiation suppressed these effects. Our results also showed that Bcl-xL and Bcl-2 levels decreased after UVB exposure, whereas Bax expression level remained unchanged by UVB exposure, resulting in a decrease in Bcl-xL/Bax or the Bcl-2/Bax ratio. These results suggest that the change in the Bcl-xL/Bax or Bcl-2/Bax ratio after UVB irradiation was involved in mitochondrial dysfunction. As shown in Figure 6A, afzelin treatment after UVB irradiation led to early recovery of the UVB-induced change in Bcl-xL/Bax or the Bcl-2/Bax ratio. Alterations in mitochondrial integrity and function may play an important role in the apoptotic cascade. The mitochondrial permeability transition (MPT), which is associated with the opening of large pores in mitochondrial membranes, is a very important event in apoptosis, and ROS are one of the major stimuli that change the MPT [27]. We examined the changes in the mitochondrial membrane potential and the MPT using the JC-1 fluorescent probe to determine whether afzelin modulates the MPT after exposure to UVB. UVB-induced disruption of the mitochondrial membrane potential and MPT was attenuated in afzelin-treated cells after UVB irradiation compared to that in untreated control cells (Figure 6B). The levels of intracellular peroxides in the HaCaT cell mitochondria were evaluated by fluorometer with the oxidant-sensitive probe DHR123 to determine whether changes in the MPT were accompanied by changes in intracellular ROS. As shown in Figure 6C, fluorescence intensity was significantly lower in cells treated with afzelin after UVB irradiation when compared to that in mitochondria of untreated HaCaT cells after exposure to UVB. These results indicate that UVB most likely leads to increased mitochondrial injury, and that afzelin protected mitochondria from oxidative damage. Next, using mitochondrial fractionation and ELISA, we determined the release of mitochondrial cytochrome *c* into the cytosol, a hallmark of intrinsic pathway-mediated apoptosis. Mitochondria play an important role regulating apoptosis by releasing apoptogenic molecules including cytochrome *c* [27]. When irradiated with an apoptosis-inducing dose of UVB (20 mJ/cm^2^), HaCaT cells displayed increased cytochrome *c* levels in the cytoplasmic fraction 12 h after UVB irradiation. A corresponding decrease in mitochondrial cytochrome *c* levels was observed after UVB irradiation. However, treatment of cells with afzelin after UVB irradiation resulted in a significantly reduced release of mitochondrial cytochrome *c* (Figure 6D). These data suggest that in addition to the UV-absorbing activity, afzelin exerts an anti-apoptotic effect against UV irradiation.

### Afzelin Inhibits UVB-induced MAPK Protein Phosphorylation

**Figure 7:**
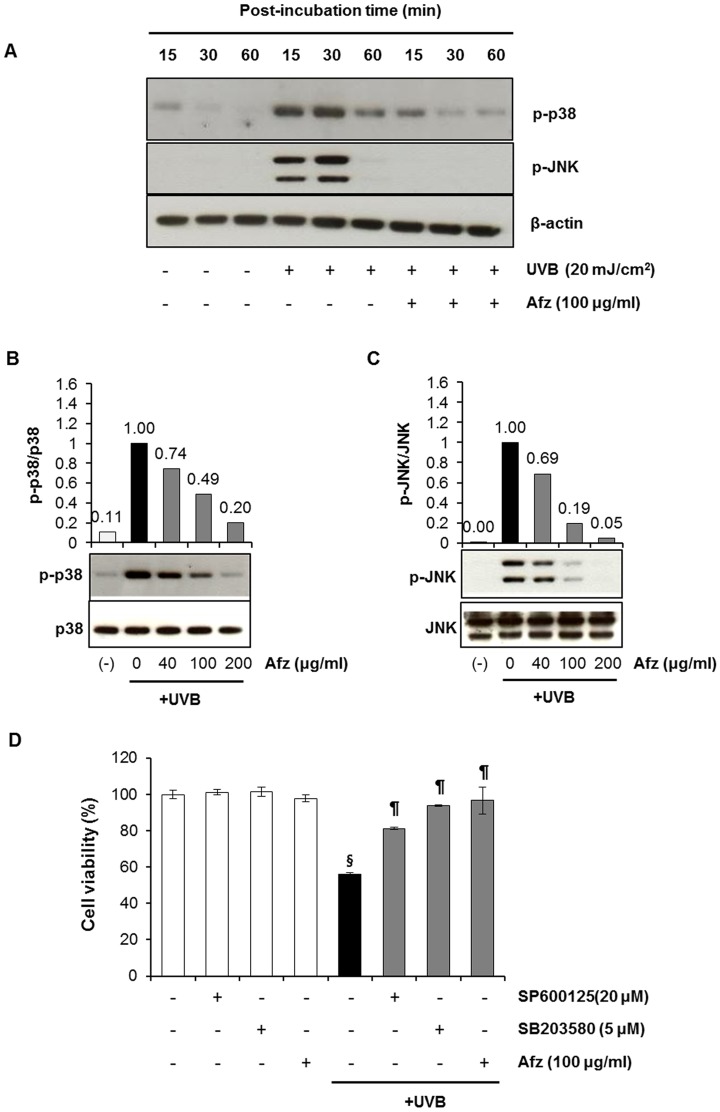
Afzelin treatment protects HaCaT cells against the UVB-induced decrease in cell viability by inhibiting p38 MAPK and JNK activity. **A.** HaCaT cells were irradiated with 20 mJ/cm^2^ UVB and subsequently treated with afzelin (100 µg/ml) for the indicated times. Total cell lysates were prepared and subjected to immunoblot analysis. Bands for phospho-p38 MAPK, and phospho-SAPK/JNK were detected and normalized to β-actin. **B** and **C.** HaCaT cells were irradiated with 20 mJ/cm^2^ UVB and treated for 30 min with various concentration of afzelin (0–200 µg/ml). Total cell lysates were prepared at 30 min after irradiation and subjected to immunoblot analysis. The bands for phospho-p38 MAPK (**B**), and phospho-SAPK/JNK (C) were detected and normalized to their total forms. The relative density of each band after normalization is shown under each immunoblot as a fold-change compared with that of the UVB-exposed control, which was assigned an arbitrary unit of 1. Representative data, n = 3 (A–C). **D.** HaCaT cells were exposed to UVB (20 mJ/cm^2^) and treated with SB203580 (5 µM), SP600125 (20 µM), and afzelin (100 µg/ml) for 24 h. Cell viability was analyzed by the MTT assay. Data are means ± SD, n = 3 (D). ^§^ *P*<0.01 compared with the vehicle-treated group, ^¶^ *P*<0.01 compared with the UVB treated group (D).

UVB-induced oxidative stress has been implicated in MAPK protein phosphorylation. Therefore, we determined the effects of afzelin on UVB-induced phosphorylation of ERK1/2, JNK, and p38 MAPK proteins in HaCaT cells. We treated HaCaT cells for 1 h with afzelin (40–100 µg/ml) after irradiation with 20 mJ/cm^2^ UVB. The cells were harvested 15–60 min after irradiation, and phosphorylated ERK, JNK and p38 MAPK expression was analyzed. Maximum increases in JNKs and p38 MAPK phosphorylation were detected 30 min after UVB (20 mJ/cm^2^) exposure (Figure 7A). Afzelin suppressed UVB-induced phosphorylation of p38 MAPK (Figure 7B) and JNKs (Figure 7C) in a concentration-dependent manner in HaCaT keratinocytes. However, we did not detect UVB-induced ERK1/2 phosphorylation. This was consistent with the finding that ERK is generally activated by growth factors and cytokines, whereas p38 MAPK and JNK are activated by stress-sensitive pathways [28].

We subsequently investigated whether the MAPK signaling pathway is involved in the photoprotective effects of afzelin. We treated HaCaT cells for 30 min with kinase inhibitors, such as SB203580 (p38 MAPK inhibitor) or SP600125 (JNK inhibitor), after irradiation with 20 mJ/cm^2^ UVB. The effect of SB203580 and SP600125 on UVB-induced cell death was determined by the MTT assay. Treatment with SB203580 or SP600125 led to a significant decrease in UVB-induced cell death as determined by the MTT assay (Figure 7D). These results indicate that afzelin exerts photoprotective effects by downregulating p38 MAPK and JNK.

### Afzelin Inhibits UVB-induced Inflammation by Suppressing the p38 MAPK and JNK Pathways

**Figure 8:**
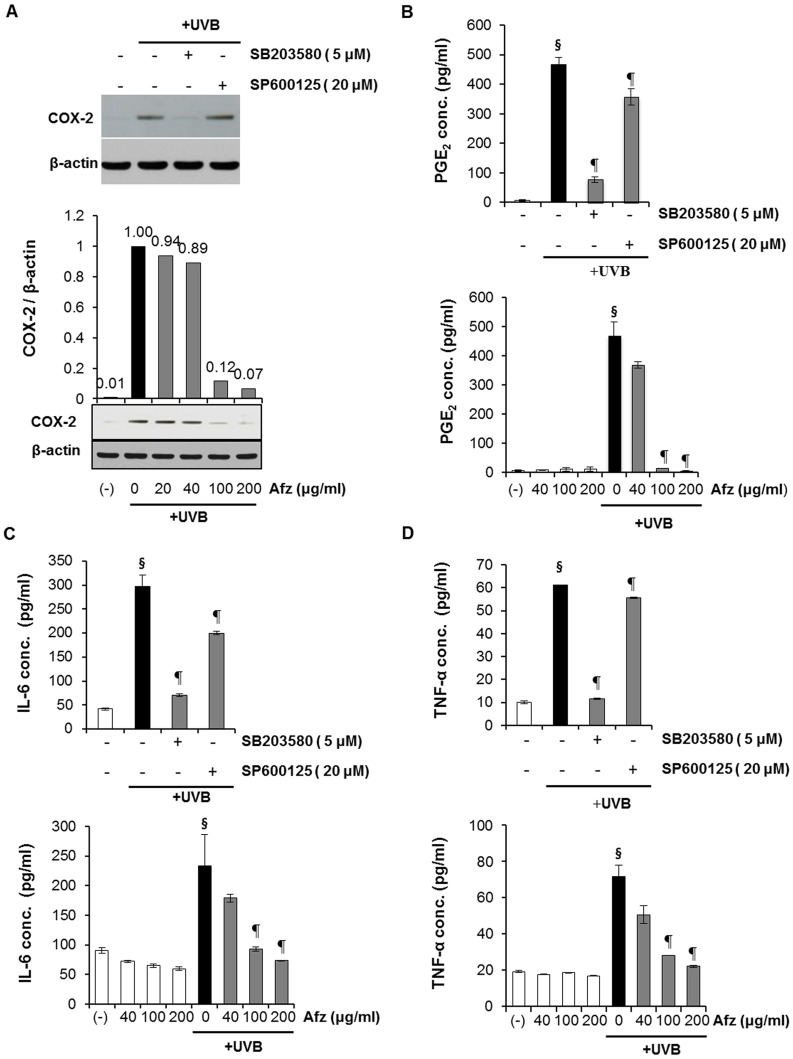
Inhibition of p38 mitogen activated protein kinase (MAPK) suppresses UVB-mediated induction of inflammatory cytokine production in human HaCaT keratinocytes. HaCaT cells were treated with the test substances (5 µM SB203580, 20 µM SP600125, or 40–200 µg/ml afzelin) after irradiation with 20 mJ/cm^2^ UVB. **A.** After 12 h, cyclooxygenase (COX)-2 protein expression was assessed by Western blotting. The histogram depicting relative COX-2 protein expression levels was normalized to β-actin expression level. Representative data, n = 3 (A). **B.** Prostaglandin-E_2_ (PGE_2_)_,_ **C.** interleukin-6 (IL-6), and **D.** tumor necrosis factor-α (TNF-α) concentrations in cell culture supernatants from the same experiments. Data are means ± SD, n = 5 (B–D). ^§^ *P*<0.01 compared with the vehicle-treated group, ^¶^ *P*<0.01 compared with the UVB-treated group (B–D).

Abnormal upregulation of COX-2 and inflammation play an important role in skin cancer [29]. Studies have shown that UVB irradiation leads to MAPK activation [30] and triggers increased COX-2 expression, which catalyzes the formation of proinflammatory prostaglandins (e.g., PGE_2_) from arachidonic acid [31], [32]. Here, we investigated whether afzelin modulated COX-2 expression in UVB-irradiated HaCaT keratinocytes. As shown in Figure 8A, UVB-induced COX-2 expression clearly decreased following treatment with the p38 MAPK inhibitor (SB203580) and afzelin. However, the effects of the JNK inhibitor (SP600125) on UVB-induced COX-2 expression were not higher than those of SB203580 treatment. These results indicate that COX-2 expression is more dependent on p38 MAPK than JNK and that afzelin inhibited UVB-induced COX-2 expression by suppressing p38 MAPK and JNK. In addition, we consistently found that while UVB-induced production of PGE_2_ decreased significantly following afzelin and SB203580 treatment after UVB irradiation, the effects of SP600125 were not greater than those of afzelin and SB203580 (Figure 8B). The anti-inflammatory effects of afzelin were further evaluated by analyzing UVB-induced production of pro-inflammatory cytokines such as IL-6 and TNF-α. UVB (20 mJ/cm^2^) irradiation markedly upregulated IL-6 and TNF-α, which were suppressed by treatment with afzelin, the p38 MAPK inhibitor, and the JNK inhibitor after UVB irradiation (Figure 8C and 8D). However, similar to COX-2 expression and PGE_2_ production, our results show that the contribution of SP600125, the JNK inhibitor was not higher than that of afzelin and SB203580, the p38 MAPK inhibitor. Taken together, these results suggest that in addition to the UV-absorbing properties, afzelin has anti-inflammatory activity by attenuating the UVB-induced activation of p38 MAPK and JNK in HaCaT cells.

## Discussion

UV radiation generates ROS, such as singlet oxygen, superoxide radical, hydroxyl radicals, and hydrogen peroxide, in a variety of cells [33], [34]. These ROS have the potential to damage cellular components such as lipid membranes, mitochondria, proteins, and DNA and eventually result in physical and chemical damage to tissues and cell death [35], [36]. To combat the deleterious effects of UV radiation, plants and animals are equipped with various protective molecules, e.g., UV-absorbing melanin, carotenoids, and retinoids; the antioxidants ascorbic acid and tocopherol [4]; and proteins involved in DNA repair and ROS detoxification [37]. However, these endogenous protective systems may be overwhelmed by prolonged and repeated UV exposure, so additional photoprotection is required.

We showed that afzelin absorbed UV radiation and reduced UV transmission. The absorbance data showed that afzelin absorbed at 280–376 nm. The resulting reduction in photons reaching the epidermis represents a first line of defense. Plant polyphenols absorbing in the UVB and UVA range protect human skin from UV induced damage [38]. Thus, afzelin could be very effective as a natural UV filter.

Second, afzelin prevented UVB-induced CPD formation in human HaCaT keratinocytes by absorbing UVB. It has been shown that UVB exposure of <1 minimum erythemal dose is sufficient to cause DNA damage in human skin cells [39], [40]. The inhibition of CPD formation by afzelin that we showed here is important because accumulation of DNA damage may lead to p53 mutations and contribute to the development of nonmelanoma skin cancer [41]. Similar protective effects have been shown for green tea polyphenols (GTP), the most extensively studied polyphenol [42]. Topical treatment of human skin with GTP reduces UV-induced DNA damage, p53 expression, and apoptosis in human keratinocytes and human skin equivalents [43]-[45]. However, these effects were not directly due to UV absorption or sunscreen effects, because topical treatment with GTP does not prevent the formation of CPD immediately after UVB irradiation. Only in skin samples obtained 24 h after UVB exposure were CPD-positive cells significantly reduced or repaired in treated versus untreated mice [46]. In contrast, afzelin prevented UVB-induced CPD formation directly after UVB irradiation, which was probably due to its UV-absorbing effect.

Besides UV-absorbing properties, we tried to investigate cellular activities of afzelin by treating cells with afzelin after UV irradiation. We examined the intracellular ROS scavenging effect of afzelin. Not only did afzelin reduce UVB-induced oxidative stress but it also displayed a cytoprotective effect in H_2_O_2_-treated cells, showing the marked antioxidant properties of afzelin. We also examined the apoptotic pathway initiated by UVB irradiation in HaCaT cells. A perturbation in redox status, as reflected by the modulation of intracellular ROS generation, MPT alterations, caspase-3 activation, cleavage of caspase target proteins, and DNA fragmentation, was observed. However, afzelin treatment significantly improved redox status and inhibited the entire apoptotic pathway. These results indicate that the antioxidant effects of afzelin may be responsible for its anti-apoptotic effect against UV irradiation.

Apoptosis is a cell suicide process with characteristic morphological features that include nuclear membrane breakdown, chromatin condensation and fragmentation, and apoptosis [47]. Many apoptotic stimuli such as UV radiation, TNF-α, Fas ligand, and chemotherapeutic agents induce cell death by activating caspases [48]. Bcl-2 is a member of the large Bcl-2 family and protects cells from apoptosis. Bcl-2 is found on the cytoplasmic face of the outer mitochondrial membrane, nuclear envelope, and endoplasmic reticulum, and other Bcl-2 family members either exist on one or more of these membranes or associate there during apoptosis [49]. Several studies have suggested that Bax appears to permeabilize the outer mitochondrial membrane, allowing efflux of apoptogenic proteins [50]-[52]. Bax binds to the mitochondrial membrane and induces cytochrome *c* release, which subsequently activates caspase-9 and caspase-3 leading to downstream apoptotic responses [47]. The ratio between antiapoptotic (Bcl-2) and proapoptotic (Bax) protein has been suggested as a primary event determining susceptibility to apoptosis by maintaining mitochondrial integrity and inhibiting activation of the caspase cascade. The changes casused by UV irradiation are compatible with mitochondrial failure, encompassing generation of ROS and accumulation of rhodamin 123 which reflect mitochondrial swelling or changes in the mitochondrial inner membrane. A clear suppression of such damages indicates that afzelin prevents a deterioration of bioeneragetic state. In our study, afzelin treatment after UVB irradiation recovered the changed Bcl-xL/Bax and Bcl-2/Bax ratio induced by UVB, consequently suppressing UV-caused cell death in HaCaT keratinocytes. The finding that afzelin protected cells from apoptotic cell damage appears to be applicable to protection of keratinocytes, which are the primary cell type in the epidermis and play a key role in the body’s initial line of defense.

Afzelin also inhibited UVB-induced COX-2 expression and production of pro-inflammatory cytokines in HaCaT cells. We showed that these effects, at least in part, were mediated by interference with the MAPK signaling pathway. COX-2 overexpression has been implicated in the development of nonmelanoma skin cancer [53]–[55]. Importantly, topical application of polyphenols such as apigenin, silymarin, and GTP to a mouse model of carcinogenesis reduces chemical and UVB-induced tumor formation [56]–[58]. Therefore, our results suggest that afzelin has the potential to suppress skin cancer.

The MAPK pathways play crucial roles mediating extracellular stimuli and intracellular signals, which trigger cellular events including proliferation, differentiation, and apoptosis [59]. UVB activates both the JNK and p38 MAPK pathways in a variety of cell types. We found that UVB-exposure activated the JNK and p38 MAPK signaling pathways, and that afzelin treatment decreased JNK and p38 MAPK phosphorylation in UVB-exposed cells. In addition, modulation of cytokines, ROS scavenging, and blockage of UV-induced oxidative damage are dependent on the regulation of JNK and p38 MAPK activities. Therefore, our results suggests that afzelin inhibited UVB-induced oxidative stress by downregulating JNK and p38 MAPK.

The data presented here suggest that afzelin displays some of its pharmacological effects via its antioxidant properties. However, alternative explanations for the mechanisms by which afzelin alters at least some of the reported outcome measures should be considered. For example, afzelin may not reduce superoxide production per se but rather absorb penetrating UV radiation; thus, intercepting the process before the source of superoxide is upregulated. If afzelin absorbs penetrating UV, then intracellular ROS generation and further downstream events would not occur. Furthermore, H_2_O_2_ readily diffuses across cell membranes, so the intervention experiments with H_2_O_2_ may represent either primary intracellular scavenging of diffusible H_2_O_2_ or secondary events leading to additional antioxidant production. Although the measured outcomes may appear similar, this does not mean that the mechanisms of inhibition are similar or consistent. Thus, in one case afzelin may absorb UV, whereas in another case it may act as a radical sink or help restore redox balance of critical thiols, both of which would produce the observed outcomes. Finally, phenolic botanicals such as afzelin modulate cell signaling and other cellular processes independent of their ability to react with oxidants [60].

We suggest that afzelin may be useful as an active component in dermatological formulations to support repair and regeneration of UV-irradiated skin. Development of broad-spectrum protective agents may help to prepare more effective sunscreens with better protection. Our results reveal that afzelin reduced UVB and UVA-induced damage to keratinocytes. Afzelin seems to be a promising candidate to photoprotect skin against UVB-induced damage and may also be efficient against UVA irradiation, as shown here. However, skin is a complicated organ consisting of several layers and cell types that influence each other during and after irradiation. Thus, further research is needed to specify the effects of afzelin on other skin cells such as fibroblasts and melanocytes and to define its effects and safety in animal models and humans.

**Figure 9:**
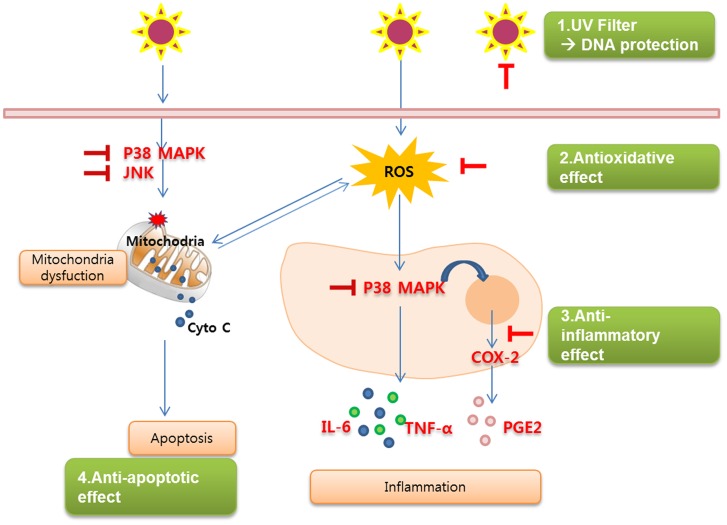
Schemic summary of the complex skin-protecting properties of afzelin. Afzelin protects the skin from the deleterious effects of UVB by exerting cellular activities (DNA protective, antioxidative, and anti-inflammatory) as well as absorbing UV.

In summary, our results suggest that afzelin protected human keratinocytes from the deleterious effects of UV irradiation through its biological properties (DNA-protective, antioxidant, and anti-inflammatory) as well as acting as a UV absorber (Figure 9). Thus, afzelin may prevent photoaging and the development of skin cancer.
